# JAZF1 heterozygous knockout mice show altered adipose development and metabolism

**DOI:** 10.1186/s13578-021-00625-1

**Published:** 2021-08-19

**Authors:** Jain Jeong, Soyoung Jang, Song Park, Wookbong Kwon, Si-Yong Kim, Soyoen Jang, Jiwon Ko, Si Jun Park, Su-geun Lim, Duhak Yoon, Junkoo Yi, Sanggyu Lee, Myoung Ok Kim, Seong-Kyoon Choi, Zae Young Ryoo

**Affiliations:** 1 Digestive Diseases Section, Department of Internal Medicine, Yale University School of Medicine, New Haven, CT USA; 2 Core Protein Resources Center, DGIST, Daegu, 42988 Republic of Korea; 3 Department of Brain and Cognitive Sciences, DGIST, Daegu, Republic of Korea; 4 School of Life Sciences, BK21 FOUR KNU Creative BioResearch, Kyungpook National University, Daegu, 41566 Republic of Korea; 5 Division of Biotechnology, DGIST, Daegu, Republic of Korea; 6 Department of Animal Science, Kyungpook National University, Daegu, 37224 Republic of Korea; 7 Gyeongsangbukdo Livestock Research Institute, Yeongju, Republic of Korea; 8 School of Animal Science and Biotechnology, Kyungpook National University, Daegu, Korea

**Keywords:** Adipocyte differentiation, Adipogenesis, Glucose homeostasis, Insulin resistance, JAZF1, PPARγ

## Abstract

**Background:**

Juxtaposed with another zinc finger protein 1 (JAZF1) is associated with metabolic disorders, including type 2 diabetes mellitus (T2DM). Several studies showed that JAZF1 and body fat mass are closely related. We attempted to elucidate the JAZF1 functions on adipose development and related metabolism using in vitro and in vivo models.

**Results:**

The JAZF1 expression was precisely regulated during adipocyte differentiation of 3T3-L1 preadipocyte and mouse embryonic fibroblasts (MEFs). Homozygous JAZF1 deletion (JAZF1-KO) resulted in impaired adipocyte differentiation in MEF. The JAZF1 role in adipocyte differentiation was demonstrated by the regulation of PPARγ—a key regulator of adipocyte differentiation. Heterozygous JAZF1 deletion (JAZF1-Het) mice fed a normal diet (ND) or a high-fat diet (HFD) had less adipose tissue mass and impaired glucose homeostasis than the control (JAZF1-Cont) mice. However, other metabolic organs, such as brown adipose tissue and liver, were negligible effect on JAZF1 deficiency.

**Conclusion:**

Our findings emphasized the JAZF1 role in adipocyte differentiation and related metabolism through the heterozygous knockout mice. This study provides new insights into the JAZF1 function in adipose development and metabolism, informing strategies for treating obesity and related metabolic disorders.

**Supplementary Information:**

The online version contains supplementary material available at 10.1186/s13578-021-00625-1.

## Background

A recent genome-wide association study indicated that new variants in gene loci modestly affect the risk of type 2 diabetes mellitus (T2DM) and related metabolic disorders. Here, variants from six genes, namely, JAZF1, TSPAN8, THADA, ADAMTS9, CDC123/CAMK1D, and NOTCH2, are genes closely associated with T2DM and metabolic disorders [1].

Several reports show that genetic variants within JAZF1 cause metabolic disorders that downregulate JAZF1 expression, and these genetic variations are also correlated with decreased body mass index (BMI) and waist circumference [1, 2]. Studies assessing the JAZF1 role in glucose and lipid metabolism and showed that JAZF1 overexpression reduced adipocyte differentiation in 3T3-L1 preadipocyte in vitro and mentioned that JAZF1 is a negative regulator in adipocyte differentiation [11–14]. Nevertheless, these data indicated that JAZF1 expression was paradoxically upregulated with adipocyte differentiation markers. Moreover, JAZF1, which promotes visfatin expression in mature adipocytes, was mediated by the peroxisome proliferator-activated receptor α (PPARα) and PPARβ/δ [3].

Interestingly, in our preliminary analyses in human adipose tissue from obese subjects, we found that increased JAZF1 expression in several microarray-based mRNA expression profiles (GSE2508, GSE9624, and GSE16415, Additional file 1: Figure S1a–c) [4–6]. A recent study demonstrated that JAZF1 represses nuclear receptor subfamily 2, group C, member 2 (NR2C2)-mediated transcription in vitro through their interaction [7]. Moreover, the NR2C2-knockout mice showed decreased adipose development, obesity-related inflammation, hepatic steatosis, and insulin resistance [8]. Thus, JAZF1 might influence metabolic regulation, adipose development, adipocyte differentiation, obesity, and insulin resistance through indirectly NR2C2-mediated transcriptional regulation.

We used the 3T3-L1 preadipocyte—a widely used model for adipogenic differentiation and mouse embryonic fibroblasts (MEFs) from the homozygous JAZF1 deletion (JAZF1-KO) mice—to explore the JAZF1 function in adipocyte differentiation related to metabolic disorders. Besides, the JAZF1 role in adipocyte differentiation, adipose tissue mass, and metabolic regulation was examined with heterozygous JAZF1 deletion (JAZF1-Het) mice fed a normal diet (ND) or a high-fat diet (HFD). The in vitro and in vivo models of JAZF1 deficiency allowed us to investigate the relationship between JAZF1 and adipose development and metabolism, helping us better understand the specific JAZF1 functions.

## Results

### JAZF1 expression during 3T3-L1 preadipocyte differentiation

3T3-L1 preadipocytes have been widely used as an in vitro model for adipocyte biology investigation, including adipocyte differentiation (i.e., adipogenesis) and lipid metabolism [12–14]. By using the 3T3-L1 model, we evaluated the JAZF1 mRNA expression during the preadipocyte differentiation, which can be divided into two stages: the early (days 0–2) and terminal stage (days 2–8) [9].

**Fig. 1:**
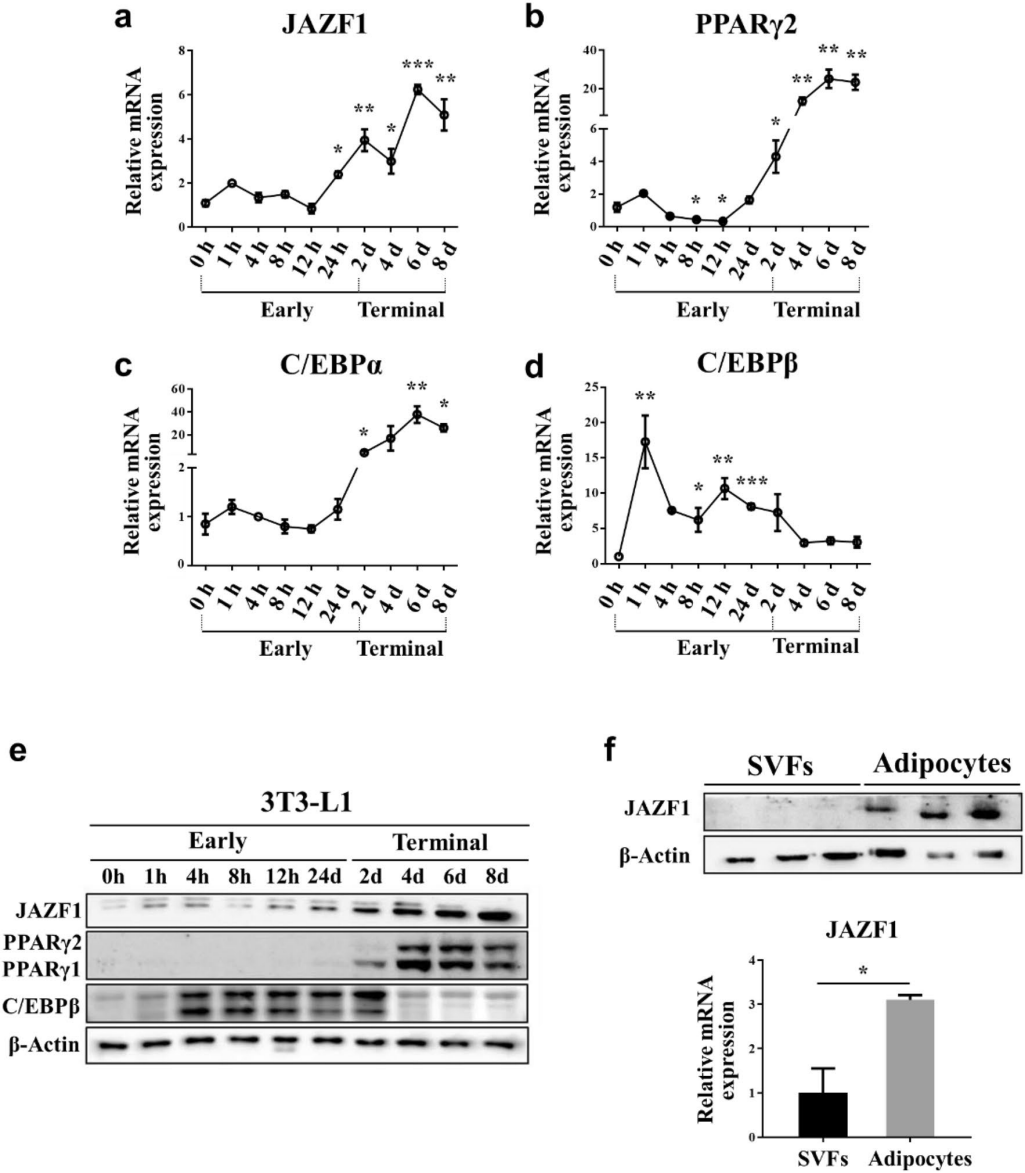
JAZF1 expression is regulated during 3T3-L1 preadipocyte differentiation. Quantitative real time polymerase chain reaction (qRT-PCR) time-course analyses of the relative **a** JAZF1, **b** PPARγ2, **c** C/EBPα, and **d** C/EBPβ mRNA expressions during early (0–2 days) and terminal (2–8 days) adipocyte differentiation in 3T3-L1. Results are representative of three independent experiments. **e** Western blot time-course analyses of JAZF1, PPARγ, and C/EBPβ protein expressions during early and terminal adipocyte differentiation in 3T3-L1. **f** JAZF1 protein (upper panel) and mRNA (bottom panel) expressions in SVFs and mature adipocytes from fractionated eWAT in wild-type C57BL/6J mice. Results (mRNA expression) are representative of three independent experiments. All data are presented as mean ± SEM. *p < 0.05, **p < 0.01, and ***p < 0.001. *SVFs* stromal vascular fractions

The JAZF1 mRNA expression was elevated mostly during the terminal adipocyte differentiation stage (Fig. 1a). We additionally checked the adipogenic markers to confirm the relation between JAZF1 and terminal differentiation. PPARγ2 and CCAAT-enhancer-binding protein α (C/EBPα) are required for adipocyte differentiation in the terminal stage [10]. The increased PPARγ2 and C/EBPα mRNA expressions are accompanied by a parallel increase in the JAZF1 mRNA expression (Fig. 1a–c). On the other hand, C/EBPβ, which rapidly induced cell proliferation during the early stage of adipocyte differentiation [11], was inversely correlated with the JAZF1 mRNA expression (Fig. 1a, d). Protein expression pattern of JAZF1 and adipogenic markers (PPARγ2, C/EBPβ) were consistent to mRNA expression levels during adipocyte differentiation (Fig. 1e). A more significantly relative JAZF1 expression is found in mature adipocytes than stromal vascular fractions (SVFs), containing preadipocytes (immature adipocytes, Fig. 1f). These observations suggest that JAZF1 may play regulatory role during adipocyte differentiation.

### JAZF1 deficiency impairs adipocyte differentiation in vitro

**Fig. 2:**
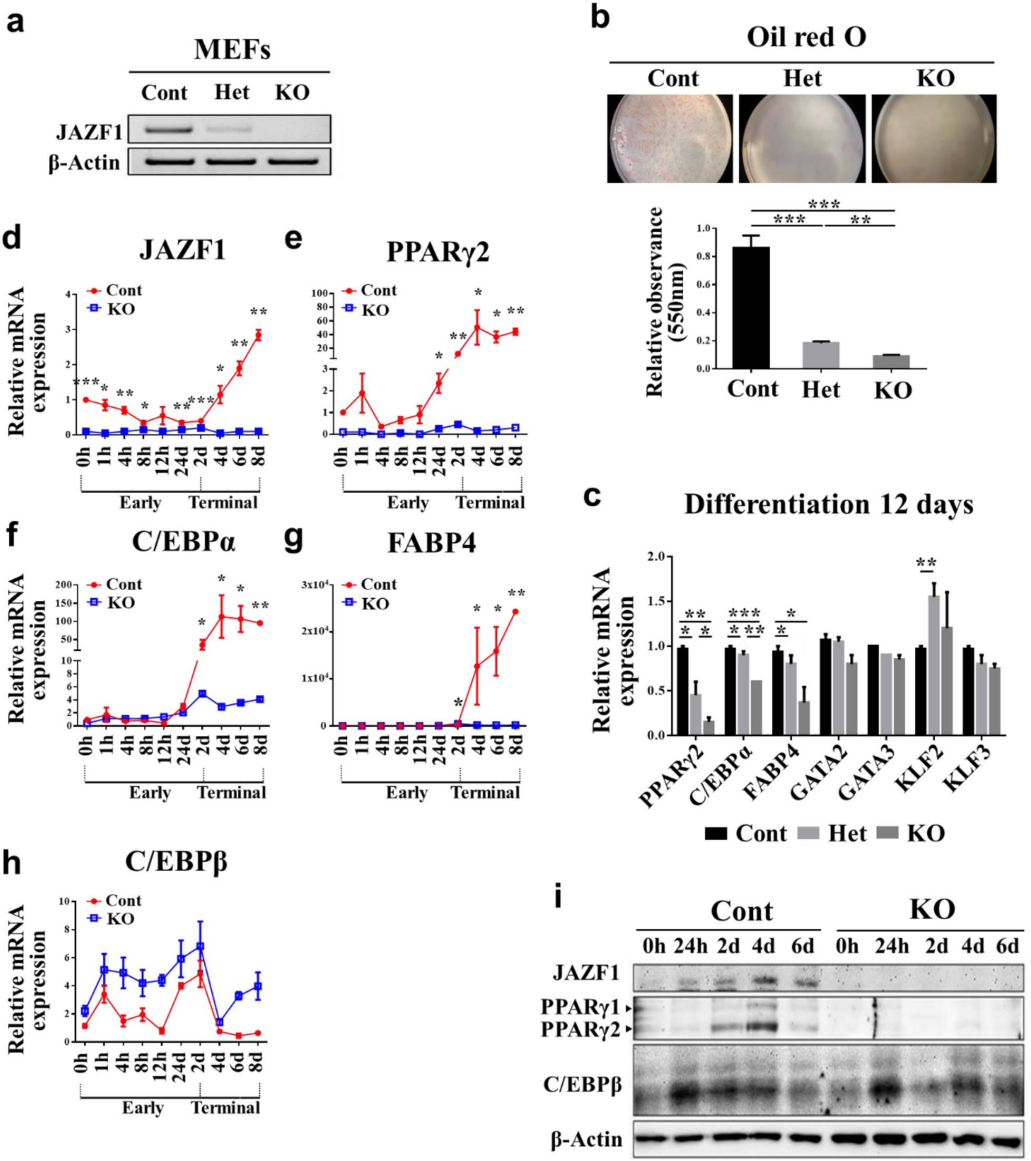
JAZF1 deficiency show impaired adipocyte differentiation in vitro. MEFs were isolated from E13.5 JAZF1-KO (KO), JAZF1-Het (Het), and JAZF1-Cont (Cont) embryos. **a** JAZF1 mRNA expressions were confirmed by semiquantitative PCR in isolated MEFs. **b** JAZF1-KO, JAZF1-Het, and JAZF1-Cont MEFs were induced adipocyte differentiation. Twelve days after induction of adipocyte differentiation, cells were stained with Oil red O. The staining was quantified spectrophotometrically at 550 nm. Results are representative of three independent experiments. **c** Relative mRNA expression of adipogenic markers after adipocyte differentiation (Day 12) in JAZF1-KO, JAZF1-Het, and JAZF1-Cont MEFs. Results are representative of three independent experiments. qRT-PCR time-course analyses of the relative **d** JAZF1, **e** PPARγ2, **f** C/EBPα, and **g** FABP4 and **h** C/EBPβ mRNA expressions during early (0–2 days) and terminal (2–8 days) adipocyte differentiation in JAZF1-KO and JAZF1-Cont MEFs. Results are representative of three independent experiments. **i** Western blot analyses of JAZF1, PPARγ, and C/EBPβ protein expressions, during adipocyte differentiation, in JAZF1-KO and JAZF1-Cont MEFs. All data are presented as mean ± SEM. *p < 0.05, **p < 0.01, and ***p < 0.001. *MEFs* mouse embryonic fibroblasts

We generated heterozygous JAZF1 deletion mice and induced adipocyte differentiation in the MEFs isolated from JAZF1-KO, JAZF1-Het, and JAZF-Cont mice embryos in vitro (Fig. 2a, b) to further investigate the JAZF1 role in adipose development and differentiation. Oil red O staining showed that JAZF1-KO MEFs treated with an adipogenic hormone cocktail failed to undergo adipocyte differentiation compared with JAZF1-Cont MEFs (Fig. 2b). JAZF1-Cont MEFs showed the highest adipocyte differentiation level, whereas the adipocyte differentiation level from JAZF1-Het MEFs was intermediate compared to those of both the JAZF1-KO and JAZF1-Cont MEFs (Fig. 2b). Consistently, the JAZF1-KO MEFs showed a profoundly reduced adipogenic marker expression during adipocyte differentiation (12 days), such as PPARγ2, C/EBPα, and fatty acid-binding protein 4 (FABP4), with severe adipocyte differentiation defect (Fig. 2c).

Next, we investigated the time-course mRNA expression of key adipogenic markers in JAZF1-KO and JAZF1-Cont MEFs. As expected, JAZF1 mRNA expression in JAZF1-Cont MEFs increased significantly during the terminal adipocyte differentiation (24 h to 8 days) (Fig. 2d). This result was consistent with the findings in 3T3-L1 preadipocyte differentiation**.** We investigated the master regulator of adipocyte differentiation, PPARγ and related genes including C/EBPα and FABP4. Unlike the JAZF1-Cont MEF, these genes were not transcriptionally active throughout adipocyte differentiation in JAZF1-KO MEFs (Fig. 2e–g). On the other hand, C/EBPβ, which encodes upstream regulator of PPARγ, was unaltered according to genotypes (Fig. 2h). Both the JAZF1 and adipogenic protein expressions also showed similar patterns, as shown in the mRNA expression level during adipocyte differentiation (Fig. 2i). Overall, JAZF1 deficiency dramatically decreased PPARγ and PPARγ-associated gene expressions.

### JAZF1-Het mice showed a lower adipose tissue mass

We generated mice with a targeted mutation at the endogenous JAZF1 locus, using the Cre-loxP system to determine the JAFZ1 involvement more deeply during adipose development and differentiation (Additional file 1: Figure S2a, b). However, the JAZF1 homozygous deletion led to embryonic lethality at E15.5–18.5 days (Additional file 1: Figure S2c). Although a few JAZF1-KO pups survived at birth, they showed severe growth retardation and died within 3 weeks (Additional file 1: Figure S2d). Therefore, we examined adipose development using JAZF1-Het mice.

**Fig. 3:**
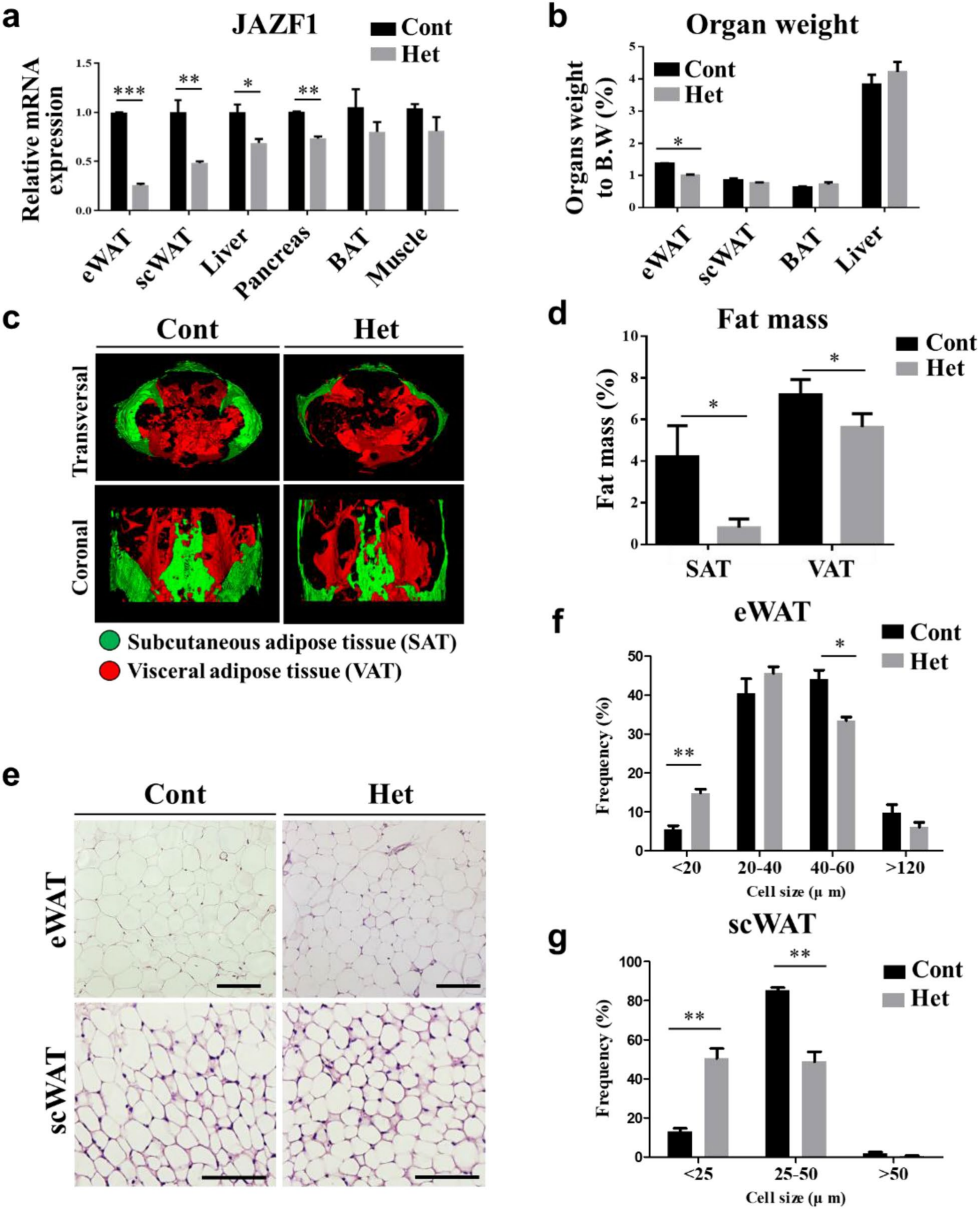
JAZF1-Het mice showed a lower adipose tissue fat mass. JAZF1-Het (Het) and JAZF1-Cont (Cont) mice were fed with ND for 8 weeks starting at 8-week-old. **a** Relative JAZF1 mRNA expression in several tissues (eWAT, scWAT, liver, pancreas, BAT, and muscle) in JAZF1-Het and JAZF1-Cont mice (8-week-old, n = 5). After 8 weeks of ND feeding, **b** PPercent of organs weight (eWAT, scWAT, BAT, and liver) were measured to total body weight (n = 5). **c** Whole-body composition analysis was performed by micro-CT in JAZF1-Het and JAZF1-Cont mice. Green areas represent subcutaneous adipose tissue (SAT) mass; the red area represents visceral adipose tissue (VAT) mass. SAT and VAT masses are presented as percentages to total body volume mass (n = 5). **d** Lean mass is presented as a percentage of total body volume mass − total adipose tissue mass (n = 5). **e** H&E staining of eWAT and scWAT in JAZF1-Het and JAZF1-Cont mice fed with ND for 8 weeks. Scale bar, 100 µm. Quantification of **f** eWAT and **g** scWAT adipocyte diameters frequency in both the JAZF1-Het and the JAZF1-Cont mice fed with ND for 8 weeks (left panel). Mean adipocyte diameter was also quantified by the calculation of each single adipocyte diameter (right panel). The diameters in eWAT and scWAT were quantified in three random images from each mouse (n = 6). All data are presented as mean ± SEM. *p < 0.05, **p < 0.01, and ***p < 0.001. *eWAT* epididymal white adipose tissue, *scWAT* subcutaneous adipose tissue, *BAT* brown adipose tissue

First, the qRT-PCR results showed that the JAZF1 mRNA expression reduced significantly in eWAT (~ 74%), scWAT (~ 52%), liver (~ 31%), and pancreas (~ 26%) of JAZF1-Het compared with JAZF1-Cont mice (Fig. 3a). The JAZF-Het and the JAZF1-Cont mice were fed with normal diet (ND) for 8 weeks starting at 8-week-old. After 8 weeks of ND feeding, the JAZF-Het mice showed a lower eWAT mass than the JAZF1-Cont mice (Fig. 3b). In the body composition analysis, both subcutaneous and visceral adipose tissue (SAT and VAT, respectively) mass were significantly lower in JAZF1-Het than JAZF1-Cont mice (Fig. 3c, d).

Next, we measured eWAT and scWAT adipocyte diameters and distribution in both the JAZF1-Het and JAZF1-Cont mice (Fig. 3e). The smaller distribution of adipocyte diameters was observed in eWAT and scWAT from the JAZF-Het mice (Fig. 3f, g). However, the nuclei density and genomic DNA contents per adipose tissue (eWAT and scWAT) depots were similar between the genotypes (data not shown).

**Fig. 4:**
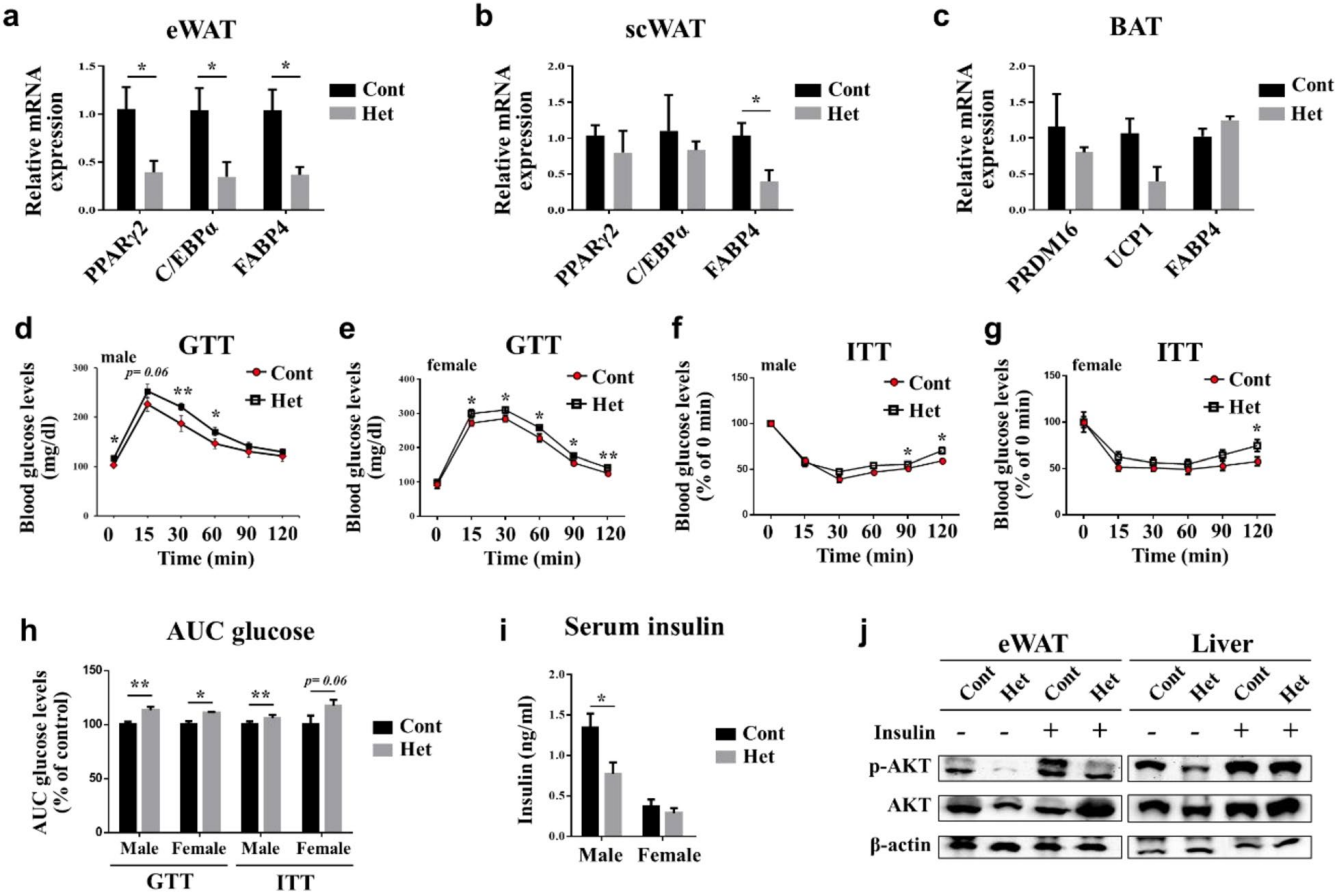
JAZF1-Het mice show impaired glucose homeostasis. JAZF1-Het (Het) and JAZF1-Cont (Cont) mice were fed with ND for 8 weeks starting at 8-week-old. After 8 weeks of ND feeding. **a**–**c** Relative mRNA expression of adipogenic markers (PPARγ2, C/EBPα, and FABP4) in eWAT, scWAT, and BAT from JAZF1-Het and JAZF1-Cont mice fed with ND for 8 weeks (n = 6). **d**, **e** GTT blood glucose levels were measured in male and female JAZF1-Het and JAZF1-Cont mice after injecting the glucose (1.5 g/kg body weight [BW], n = 8). **f**, **g** ITT blood glucose levels in male and female JAZF1-Het and JAZF1-Cont mice after injecting the insulin (2 U/kg BW, n = 8). **h** Area under the curve (AUC) of the glucose levels in male and female JAZF1-Het and JAZF1-Cont mice (n = 8 for each group), as determined by GTT and ITT glucose levels. The data is shown by percent (%) of control mice AUC glucose levels. **i** Serum insulin levels in 6 h fasted male and female JAZF1-Het and JAZF1-Cont mice after 8 weeks of ND feeding. (n = 6 or 7 for each group). **j** Western blotting of insulin-stimulated (2 U/Kg BW) AKT phosphorylation in the eWAT and liver lysates from both the JAZF1-Het and the JAZF1-Cont mice fed with ND for 8 weeks. All data are presented as mean ± SEM. *p < 0.05 and **p < 0.01. *GTT* glucose tolerance test, *ITT* insulin tolerance test, *eWAT* epididymal white adipose tissue, *scWAT* subcutaneous adipose tissue, *BAT* brown adipose tissue

We performed a qRT-PCR to analyze the molecular changes of adipose tissues [eWAT, scWAT, and brown adipose tissue (BAT)] in the JAZF1-Het mice. We found that the expressions of adipogenic markers were significantly lower in the eWAT and scWAT compared to WT mice (Fig. 4a, b). Nonetheless, as seen in Fig. 3a, JAZF1 did not change significantly in BAT and neither did the mRNA expression of BAT makers (PRDM16, UCP1, and FABP1; Fig. 4c). Besides, lipogenesis-related gene expressions in the liver decreased in JAZF1-Het compared to JAZF1-Cont mice, although histological analysis showed no difference between the two groups **(**Additional file 1: Figure S3a, b).

We also found JAZF1-Het mice had lower triglyceride (TG) levels and higher nonesterified fatty acids (NEFA) levels in serum (Additional file 1: Figure S4a, b). Serum high-density lipoprotein (HDL) levels decreased in the JAZF1-Het mice, but no difference was observed in serum low-density lipoprotein (LDL) and cholesterol level, independently of the genotype considered (Additional file 1: Figure S4c–e). Together, these results suggest that JAZF1 heterozygous deletion reduced adipose development and affected the lipid profiles in blood circulation.

### JAZF1-Het mice showed impaired glucose homeostasis but normal energy metabolism

Interestingly, the change in adipogenic markers was more significant in the eWAT compared to scWAT (Fig. 4a, b). As a visceral adipose tissue, eWAT accumulation is associated with metabolic risks, including insulin resistance. We performed glucose tolerance test (GTT) and insulin tolerance test (ITT) analysis on JAZF1-Het and JAZF1-Cont mice to determine whether a JAZF1 heterozygous deletion could change the systemic glucose homeostasis and insulin sensitivity. Male and female JAZF1-Het mice fed with ND for 8 weeks showed higher blood glucose levels than the JAZF1-Cont mice during the GTT (Fig. 4d, e). The ITT results revealed that the blood glucose levels in male JAZF1-Het mice were higher at 90 and 120 min, and female JAZF1-Het mice were higher at 120 min, following the insulin injection (Fig. 4f, g). Consistently, the area under the curve (AUC) for the glucose levels determined by GTT increased in the male and female JAZF1-Het mice compared with the JAZF1-Cont mice (Fig. 4h). Regarding the AUC glucose level determined from ITT, the male JAZF1-Het mice showed a significant increase compared with male JAZF1-Cont mice. However, female JAZF1-Het mice showed an increased trend in the AUC of the glucose level (*p* = *0.06*) compared with the female JAZF1-Cont mice. According to the genotypes, the serum insulin level in male JAZF1-Het mice was significantly lower than JAZF1-Cont mice, but no difference was observed in female mice **(**Fig. 4i). The qRT-PCR analysis showed that the mRNA expressions of insulin sensitivity markers (GLUT4, leptin, and adiponectin) were lower in both the eWAT and scWAT in the JAZF1-Het mice. However, both the gluconeogenesis and glucose homeostasis-related gene expressions in their liver did not change with JAZF1 heterozygous deletion (Additional file 1: Figure S5). We investigated insulin signaling in both the JAZF1-Het and JAZF1-Cont mice to determine organ-specific insulin action by JAZF1. We challenged the JAZF1-Het and JAZF1-Cont mice with insulin for 15 min and isolated their eWAT and liver. Western blot analysis showed that insulin-stimulated Protein kinase B (AKT) phosphorylation was significantly lower in the eWAT and liver tissues of the JAZF1-Het mice than that of the JAZF1-Cont mice (Fig. 4j).

**Fig. 5:**
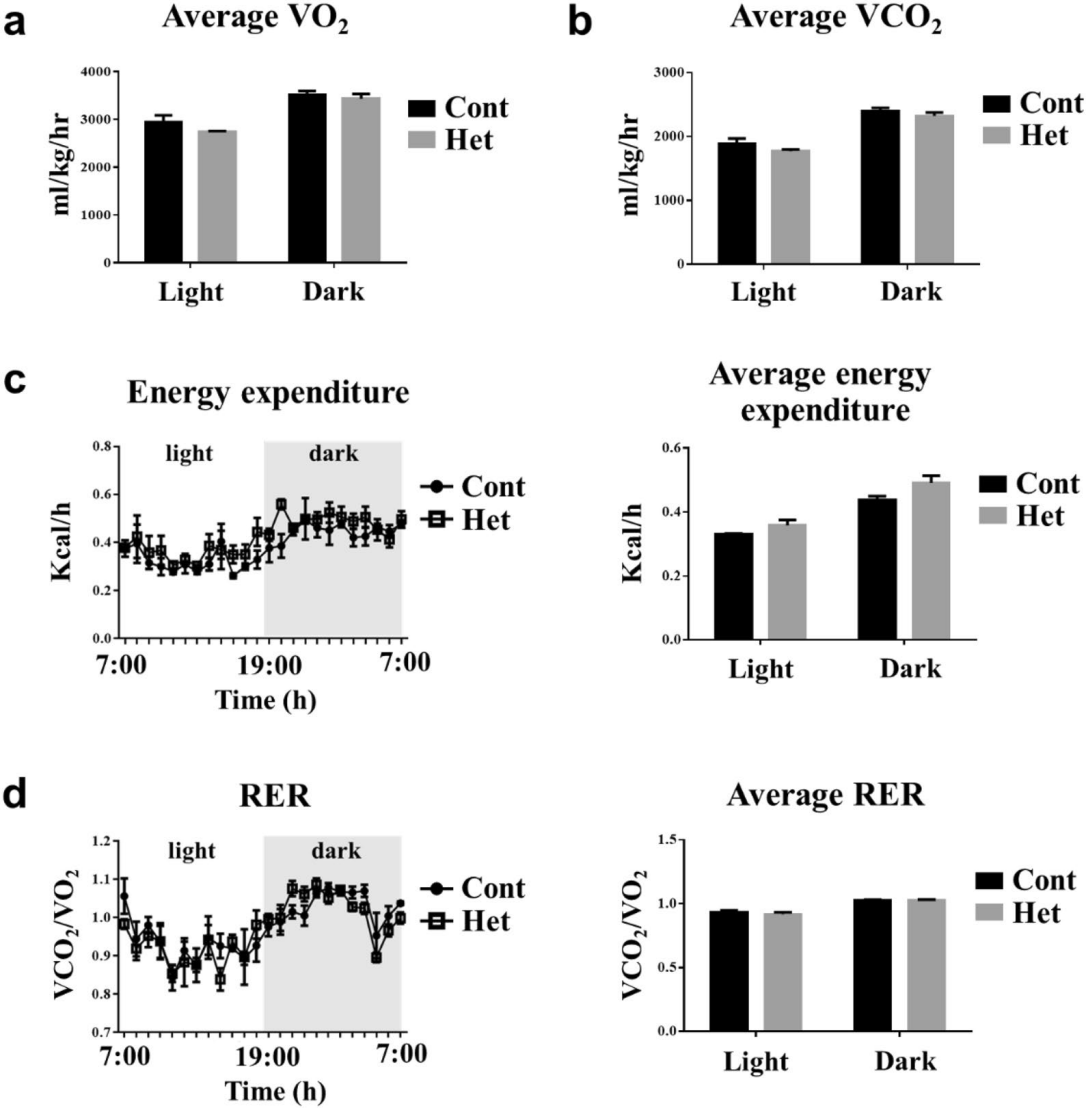
Energy metabolism analysis in both the JAZF1-Het and the JAZF1-Cont mice. The JAZF1-Het (Het) and the control (Cont) mice were fed with ND for 8 weeks starting at 8-week-old. After 8 weeks of ND feeding, **a** The average oxygen consumption and **b** volume of carbon dioxide production (VCO_2_) production were measured in both the JAZF1-Het and the JAZF1-Cont mice (n = 6). **c** Energy expenditure (EE, left panel) in JAZF1-Het and JAZF1-Cont mice fed with ND for 8 weeks (n = 6). The bar graph (right panel) represents average EE values also calculated during the day and night. **d** Respiratory exchange ratio (RER; VCO_2_/VO_2_, left panel) in JAZF1-Het and JAZF1-Cont mice fed with ND for 8 weeks (n = 6). The bar graph represents average RER values (right panel) also calculated during the day and night. All data were collected by indirect calorimetry for 24 h (7 AM to 7 PM). All data are shown as mean ± SEM

Next, we examined energy metabolism in both the JAZF1-Het and JAZF1-Cont mice by checking the amount of oxygen that their body uptakes and utilizes. Through the VO_2_ and VCO_2_, we measured the form of oxygen inhaled and exhaled by the mice (Fig. 5a, b). Moreover, we calculated both the energy consumption and respiratory exchange ratio (RER) ratio, using the indirect calorimetry system. The results show no significant difference in energy expenditure according to genoyptes (Fig. 5a–d). Therefore, the JAZF1 heterozygous deletion caused insulin resistance but did not affect energy metabolism.

### JAZF1-Het mice fed with a high-fat diet showed less adipose development and affected glucose homeostasis

**Fig. 6:**
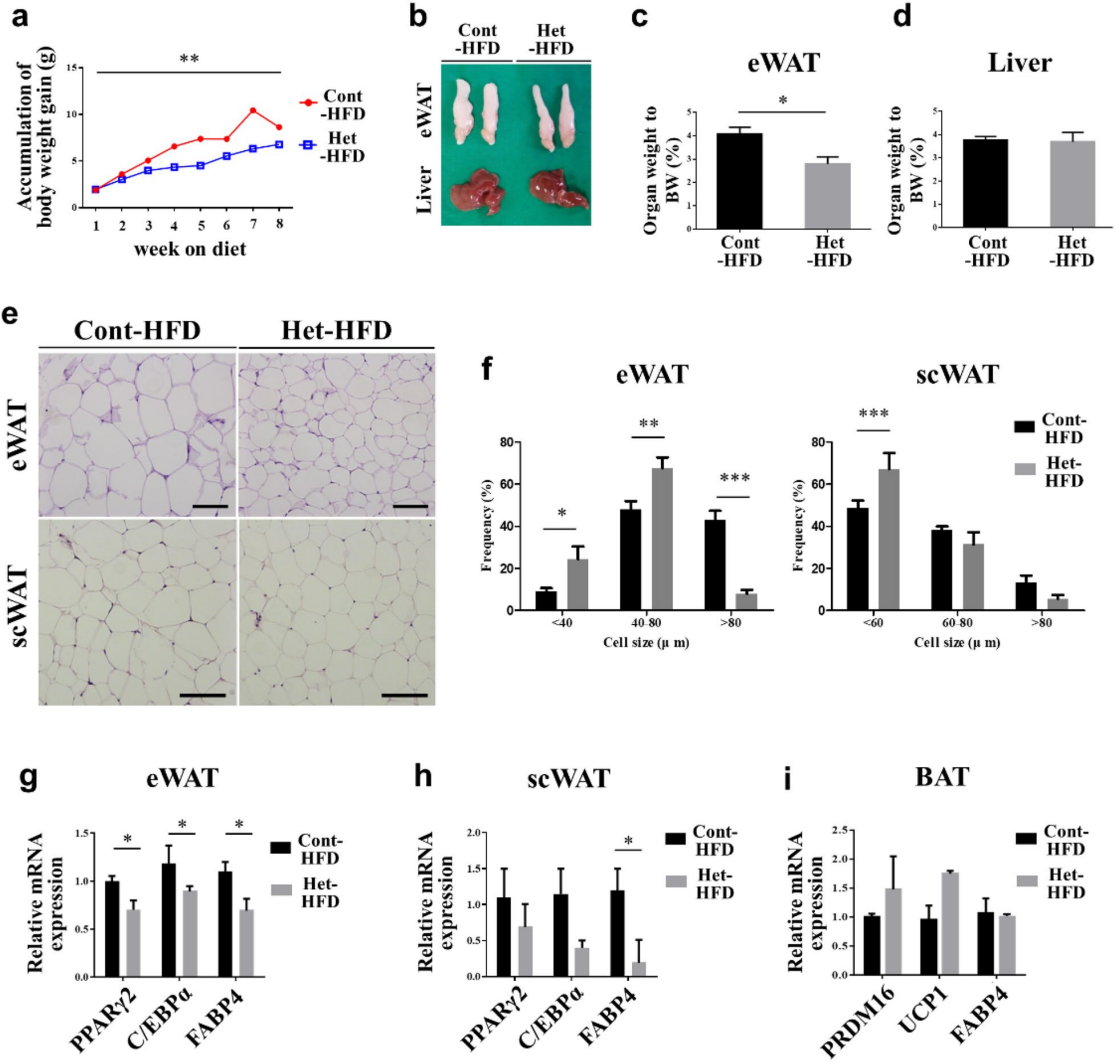
JAZF1-Het mice fed with HFD showed a lower adipose development. Eight-week-old JAZF1-Het-HFD (Het-HFD) and JAZF1-Cont-HFD (Cont-HFD) mice were fed with HFD for 8 weeks. **a** The body weight gaining accumulation during the 8 weeks of HFD feeding in both the JAZF1-Het-HFD and JAZF1-Cont-HFD mice (n = 8). **b** The morphology of eWAT and liver tissue in JAZF1-Het-HFD and JAZF1-Cont-HFD after 8 weeks of HFD feeding. **c**, **d** The percentage of eWAT and liver organ weight was measured to the total body weight (n = 7). **e** HH&E staining of eWAT and scWAT in both the JAZF1-Het-HFD and JAZF1-Cont-HFD mice. Scale bar, 100 µm. **f** Quantification of eWAT and scWAT adipocyte diameters frequency in JAZF1-Het-HFD and JAZF1-Cont-HFD mice (right panel). Mean adipocyte diameter was also quantified by calculation of each single adipocyte diameter (left panel). The diameters in eWAT and scWAT were quantified in eight random images from each mouse (n = 7). **g**–**i** Relative mRNA expression of the adipogenic marker (PPARγ2, C/EBPα, and FABP4) in eWAT, scWAT, and BAT from both the JAZF1-Het-HFD and the JAZF1-Cont-HFD mice (n = 6). All data are presented as mean ± SEM. *p < 0.05, **p < 0.01, and ***p < 0.001. *eWAT* epididymal white adipose tissue, *scWAT* subcutaneous adipose tissue, *BAT* brown adipose tissue

We fed 8-week-old JAZF1-Het and JAZF1-Cont mice with a high-fat diet (HFD) for 8 weeks (JAZF1-Het-HFD vs. JAZF1-Cont-HFD) to investigate the effect of heterozygous deletion of JAZF1 on adipose development in vivo. JAZF1-Het-HFD mice showed significantly lower weight gain than JAZF1-Cont-HFD mice (Fig. 6a). The JAZF1-Het-HFD mice also exhibited a considerably lower eWAT mass (Fig. 6b, c), and histological analysis of their adipocytes showed that both eWAT and scWAT were smaller (Fig. 6e). We calculated the distribution and mean adipocyte diameters in eWAT and scWAT. These measurements showed a significantly smaller adipocyte distribution and diameter in the JAZF-Het-HFD than in the JAZF1-Cont-HFD mice (Fig. 6f). However, the liver mass (Fig. 6d), nuclei density, and genomic DNA contents per adipose tissue depots (eWAT and scWAT) were similar between the two groups (data not shown). The lipid accumulation in the liver was lower in the JAZF1-Het-HFD than JAZF1-Cont-HFD mice (Additional file 1: Figure S6a).

Next, we performed qRT-PCR analysis to determine the molecular change of adipose tissues in JAZF1-Het-HFD. The expressions of PPARγ2 and genes encoding other adipogenic markers were significantly lower in the eWAT of JAZF1-Het-HFD mice (Fig. 6g). Although both the PPARγ2 and C/EBPα mRNA expressions did not change in the scWAT of JAZF1-Het-HFD mice, the FABP4 mRNA expression decreased significantly (Fig. 6h). However, unlike eWAT and scWAT, JAZF1-Het-HFD mice showed no change in BAT markers compared with JAZF1-Cont-HFD **(**Fig. 6i), as in the previous results (Fig. 4c). Moreover, lipogenesis-related gene expressions in the liver decreased significantly in JAZF1-Het-HFD compared to JAZF1-Cont-HFD mice **(**Additional file 1: Figure S6b). In the serum analysis, both the TG and NEFA levels remained unchanged, corroding to genotypes **(**Additional file 1: Figure S7a, b). Serum HDL land cholesterol levels decreased in the JAZF1-Het-HFD mice, but the LDL level remained unchanged **(**Additional file 1: Figure S7c–e).

**Fig. 7:**
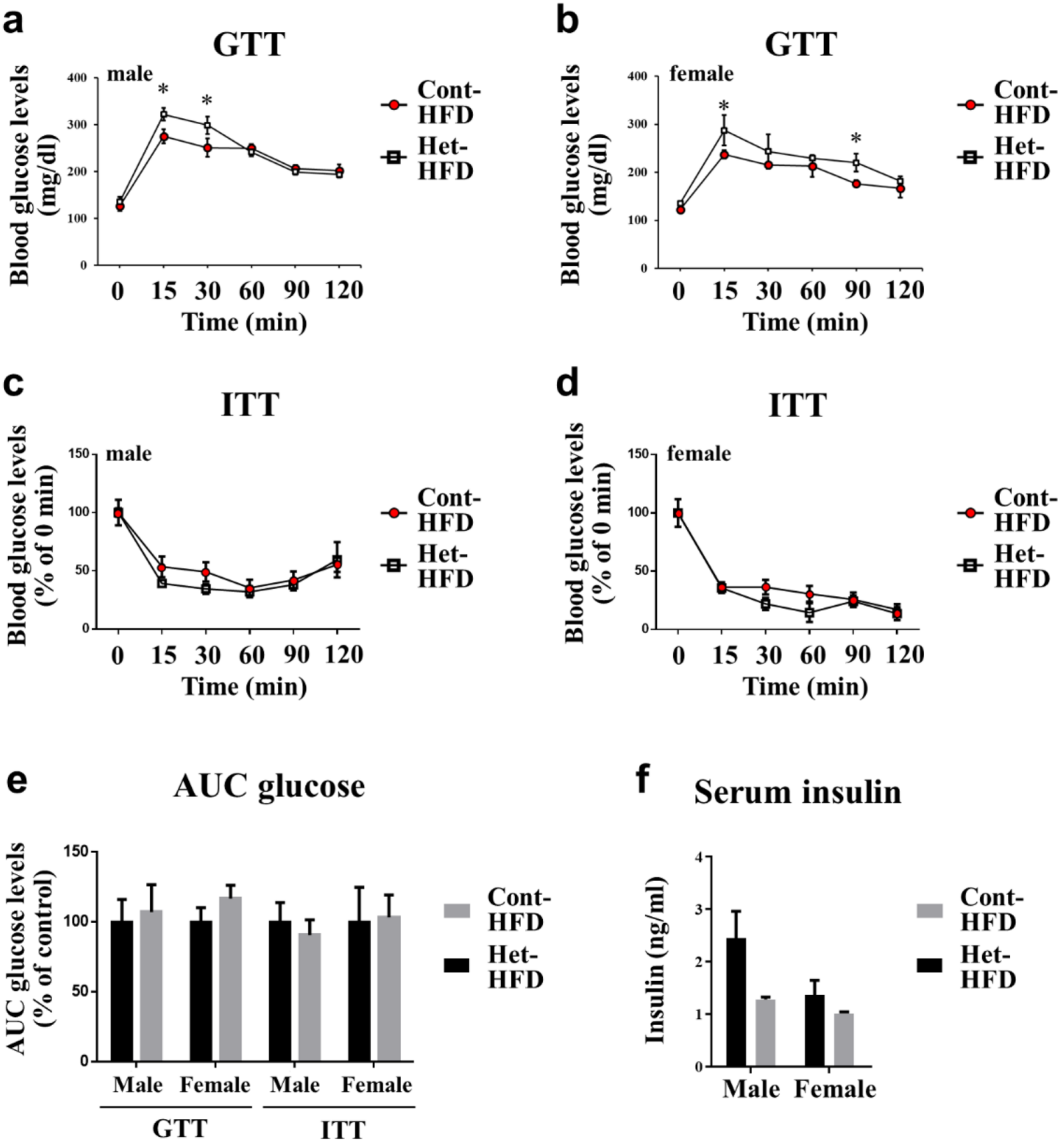
Affected glucose homeostasis in JAZF1-Het mice fed with HFD. Eight-week-old males and females of both the JAZF1-Het-HFD (Het-HFD) and JAZF1-Cont-HFD (Cont-HFD) mice were fed with HFD for 8 weeks. After 8 weeks of HFD feeding, **a**, **b** GTT glucose levels were measured in males and females of both the JAZF1-Het-HFD and JAZF1-Cont-HFD mice after injecting the glucose (1.5 g/kg body weight, n = 8). **c**, **d** ITT glucose levels in male and female JAZF1-Het-HFD and JAZF1-Cont-HFD mice after injecting the insulin (2 U/kg body weight). The data presented by percent (%) of initial (0 min) glucose levels (n = 8). **e** Area under the curve (AUC) glucose levels in males and females of both the Het-HFD and Cont-HFD mice (n = 8), as determined by GTT and ITT glucose levels. The data is shown in the percentage (%) of control mice AUC glucose levels. **f** Serum insulin levels in 6 h fasted male and female of both the Het-HFD and the Cont-HFD mice after 8 weeks of HFD feeding (n = 6). All data are presented as mean ± SEM. *p < 0.05. *GTT* glucose tolerance test, *ITT* insulin tolerance test

We also determined systemic glucose homeostasis in JAZF1-Het mice after HFD feeding for 8 weeks. The GTT results show that blood glucose levels were significantly higher in both male (at 15 and 30 min) and female (at 15 and 90 min) JAZF1-Het-HFD compared to JAZF1-Cont-HFD mice (Fig. 7a, b). However, the blood glucose level from the ITT remained unchanged according to genotypes (Fig. 7c, d). The AUC blood glucose and serum insulin levels also remained unchanged in male and female JAZF1-Het-HFD compared to JAZF1-Cont-HFD mice (Fig. 7e, f). The mRNA expressions of insulin sensitivity markers tended to decrease in eWAT and scWAT of the JAZF1-Het-HFD mice. However, gluconeogenesis and glucose homeostasis-related genes in the liver remained unchanged in the JAZF1-Het-HFD mice (Additional file 1: Figure S8).

### JAZF1-Het mice either fed with ND or HFD showed increased immune cell infiltration

**Fig. 8:**
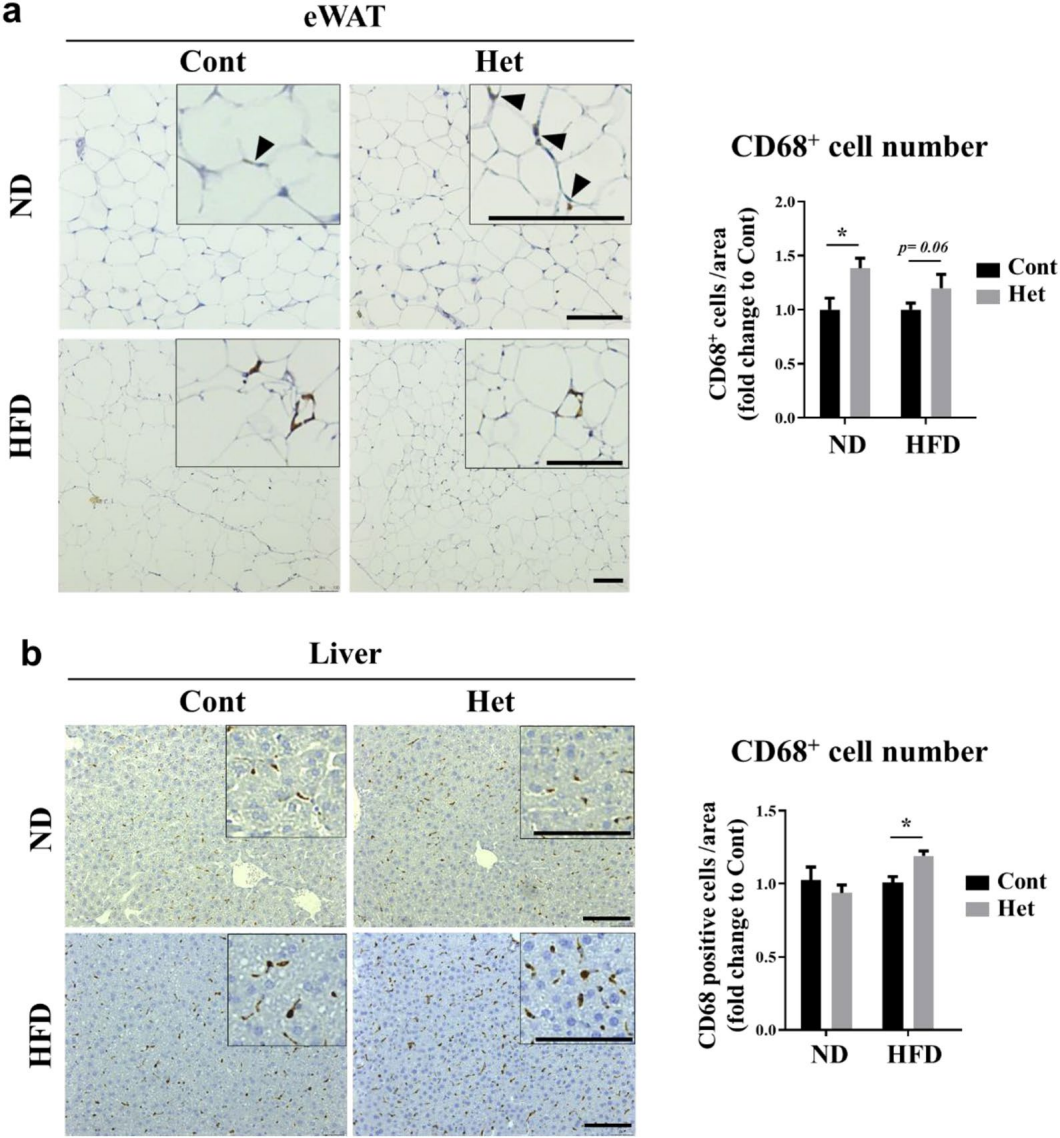
Immunohistochemical analysis of macrophages and monocytes using anti-CD68 in eWAT and liver from Het and Cont mice either fed with ND or HFD. Both the JAZF1-Het (Het) and JAZF1-Cont (Cont) mice were either fed with ND or HFD for 8 weeks. After an 8-week diet feeding either with ND or HFD, **a** representative images showing CD68-positive cells in eWAT from both the JAZF1-Het and JAZF1-Cont mice. Scale bar, 100 µm. The CD68-positive cell numbers were also quantified in eWAT (right panel). The CD68-positive cells were quantified in five random area images from each mouse (n = 7). **b** Representative images showing CD68-positive cells in the liver from both the JAZF1-Het and JAZF1-Cont mice either fed with ND or HFD for 8 weeks. Scale bar, 100 µm. The CD68-positive cell numbers were also quantified in the liver (right panel). The CD68-positive cell numbers were quantified in eight random area images from each mouse (n = 7). The black arrowheads indicate the CD68-positive cells. All data are presented as mean ± SEM. *p < 0.05. *eWAT* epididymal white adipose tissue

Immune cell infiltration in metabolic tissues is a causative factor in insulin resistance and metabolic disorders [12]. Recent studies indicate that JAZF1-overexpressing mice showed reduced chronic inflammation by regulating macrophage population and inflammatory cytokines in vivo and in vitro [13, 14]. Therefore, we determined whether heterozygous JAZF1 deletion affects the macrophage infiltrations. We either fed both the JAZF1-Het and JAZF1-Cont mice with an ND or HFD for 8 weeks. We stained the eWAT and liver tissues with CD68 antibodies. The immunohistochemical analyses showed that the number of CD68-positive macrophages and monocytes increased significantly in eWAT in the ND-fed JAZF1-Het compared to the ND-fed JAZF1-Cont mice. However, compared to the JAZF1-Cont mice, the JAZF1-Het mice fed with HFD showed a tendency to increase the number of CD68-positive cells in the eWAT tissue (Fig. 8a, p < 0.06). The CD68 positive cells in the liver did not differ between the JAZF1-Het and JAZF1-Cont mice fed with ND, but they increased in the JAZF1-Het compared to the JAZF1-Cont mice, when both groups were fed with an HFD (Fig. 8b).

## Discussion

Here, we generated heterozygous JAZF1-deficient mice and identified the JAZF1 role in adipose development and metabolism. The most critical feature caused by the JAZF1 deficiency was reducing adipose development, which led to insulin resistance. We demonstrated that JAZF1 deletion (homozygous JAZF1-deficient) inhibited adipocyte differentiation in MEFs and 3T3-L1 in vitro experimental models. Furthermore, we demonstrated that JAZF1 partially regulates adipocyte differentiation through PPARγ and JAZF1 interactions, implying that JAZF1 is extensively involved in adipose development and insulin sensitivity.

Recently, attempts have been made to find the pathological JAZF1 regulation mechanism in humans. Several single nucleotide polymorphism risk alleles within the JAZF1 gene, which led to a reduced JAZF1 expression, were associated with decreased BMI, waist circumference, and impaired glucose homeostasis [1, 2, 15–18]. The observations in our JAZF1-deficient mice are consistent with the results published in these previous reports, which were primarily performed in the JAZF1 overexpression models during adipose development [3, 13, 19–21]. Here, we used whole-body CMV-cre to delete JAZF1 in mice and demonstrated that JAZF1 deficiency inhibits adipose development in adipose tissues.

Previous in vitro and in vivo studies indicated that JAZF1 works as a negative regulator in adipose development and differentiation [3, 19–21]. Nonetheless, the JAZF1 expression, strictly regulated during adipocyte differentiation, could explain the paradoxical finding we obtained in our research. When the adipocyte is induced to differentiate, growth-arrested preadipocytes reenter the cell cycle with mitotic clonal expansion during the early differentiation stage, followed by the expression of genes that maturate the adipocytes during the terminal differentiation stage. However, in the early differentiation stages, such growth-arrested preadipocytes must exit the cell to undergo terminal differentiation [22]. The JAZF1 expression levels remained low at early differentiation in the 3T3-L1 and MEFs in our in vitro experimental models and robustly increased during the terminal differentiation.

We introduce JAZF1 as a positive regulator of adipocyte development and differentiation and we think the role of JAZF1, privously known as a negative regulator in adipocyte development and differentiation, can be explained from different perspectives. JAZF1 was originally identified as an oncogene that promotes cell cycle progression and induces cell proliferation in tumors [23, 24]. Thus, persistent JAZF1 upregulation may interrupt the exit from the cell cycle during the early differentiation stage. This process could delay or restrain the completion of differentiation and, eventually, contribute to oncogenesis. Collectively, these results provide evidence that precise JAZF1 regulation during adipocyte differentiation from the early to the terminal stage might be necessary for the process to occur.

We focused on the transcriptional regulatory aspect during the adipocyte differentiation process to elucidate the JAZF1 mechanisms during adipose development and differentiation. JAZF1 was identified as a transcription regulator and functions as a repressor of transcription of NR2C2 [7]. A study indicated that the enriched cytosolic JAZF1 that dynamically localized in nuclear upon stimulation with metabolic stress [25] may regulate the transcription activity of target nuclear receptors. In this context, a study demonstrated that JAZF1 is a positive transcriptional regulator of visfatin and promotes adipocyte differentiation and maturation [3]. Moreover, JAZF1 directly affects the transcription of ribosome biogenesis and RNA processing-related genes in β-cell [25]. Our findings expanded another JAZF1 mechanism that regulates the transactivation activity of PPARγ and adipogenic gene expressions, which are also responsible for maintaining adipocyte differentiation. To sum up, we suggesting that JAZF1 acts as a positive regulator of adipocyte differentiation through PPARγ.

However, how JAZF1 functionally interacts with PPARγ is currently unknown. One possible explanation is that JAZF1 affects adipocyte differentiation and PPARγ activity through AKT regulation—a coactivator of PPARs [26–28]. We found that JAZF1 deficiency led to decreased AKT activity in the eWAT and liver, in response to excessive insulin stimulation. This result is consistent with the finding that mice overexpressing JAZF1 show increased phosphoinositide-3-kinase/AKT activation in both the adipose tissue and liver [29].

The relationship between glucose homeostasis and JAZF1 was previously reported in the JAZF1 overexpression model. JAZF1 overexpression in transgenic mice improved insulin sensitivity and protected HFD-induced obesity [13, 21, 30]. In this study, JAZF1-deficient mice showed impaired glucose homeostasis. However, although JAZF1-deficient mice showed adiposity reduction, their insulin resistance was more developed than control mice. These observations are similar to those found previously in lipodystrophy models, in which, the resistance to lipid accumulation in dysfunctional adipose tissue causes insulin resistance with dyslipidemia [31, 32]. Compared to the control mice, the JAZF1-deficient mice had less adipose tissue mass and showed insulin resistance, with a perturbed lipid profile. However, we did not observe the development of ectopic fat accumulation, particularly in the liver of mice fed with an ND, as observed in the lipodystrophy models. One possibility is that reduced JAZF1 expression in our whole-body JAZF1-deficient mice might have inhibited fat accumulation in the liver, supported by the lower expression trend in lipogenesis-related genes in their liver. On the other hand, the inability and dysfunctional adipose tissue cause decreased secretion of insulin-sensitizing adipokines, such as leptin and adiponectin, contributing to insulin resistance. We also found reduced leptin and adiponectin mRNA expressions in both the eWAT and scWAT tissues of JAZF1-deficient mice.

We also observed an increased infiltration of monocyte and macrophage in adipose tissue and liver in JAZF1-deficient mice. Increased macrophage and immune cell infiltration is often associated with inflammation and leads to insulin resistance [12]. Indeed, JAZF1 can control the adipose tissue macrophage by regulating their antigen presentation function and populations [13, 14]. The dysregulated lipid metabolism and the change in glucose homeostasis in JAZF1-deficient mice are attributed to the role of JAZF1, in the development and differentiation of adipose tissues, which partially occurs through PPARγ. Remarkably, the inhibition of adipose development in JAZF1-deficient mice is manifested by the reduced PPARγ transcriptional activity and related genes in eWAT and scWAT, reducing lipid accumulation, maturation, and insulin-sensitizing adipokines expressions, such as leptin and adiponectin.

Our study on JAZF1-deficient mice showed that JAZF1 is involoved in metabolism, affecting fat accumulation and insulin sensitivity in metabolic-related tissues, but some limitations must be considered. Overall, the metabolic phenotypes were moderate in our heterogynous JAZF1-deficient mice. We found that fat accumulation in eWAT was inhibited in our JAZF1-deficient mice, which is correlated with the decrease in JAZF1 expression levels. However, scWAT and liver tissues showed lower metabolic changes with less decreased JAZF1 expression. Therefore, the moderate phenotypes we observed in these mice could be due to the JAZF1 downregulation efficiency. It would be better to address the JAZF1 function on adipose tissue development and metabolism by establishing a tissue-specific JAZF1 knockout mice model.

## Conclusions

We demonstrated that role of JAZF1 in adipose tissue development and related metabolism. These results support a new insight into the critical JAZF1 function in systemic metabolism. Our findings may be helpful for the treatment of metabolic diseases, such as obesity and diabetes.

## Methods

### Generation of heterozygous JAZF1 deletion mice

The gene-targeting vector with the loxP site flanking the critical exon 2 of JAZF1 was constructed by EUCOMM (European Conditional Mouse Mutagenesis Program) consortium. Heterozygous mice carrying a JAZF1^tm1a (EUCOMM)Wtsi^ allele were established from Macrogen (Seoul, Korea). The schematic procedure for generating the heterozygous JAZF1 deletion (JAZF1-Het) mice is shown in Additional file 1: Figure S2a. The offspring genotype was confirmed by PCR, using tail biopsies from homozygous JAZF1 deletion (JAZF1-KO), JAZF1-Het, and their littermate wild-type control (JAZF1-Cont) pups (Additional file 1: Figure S2b). Briefly, mice carrying the wild-type allele were confirmed using oligonucleotide primers 5ʹ-arm and 3ʹ-arm, which produced a 426-bp product. The presence of the targeted allele was confirmed using oligonucleotide primers 5ʹ-arm and LAR3, which produced a 321-bp product. Mice carrying the mutated allele produced by Cytomegalovirus (CMV)-Cre-recombination were confirmed using oligonucleotide primers Cre-forward and Cre-reverse, which produced a 641-bp product. All oligonucleotide sequences are provided in Additional file 1: Table S1.

### Animal and diet treatment

All animal experiments adhered to the guidelines for animal experimentation of the Kyungpook National University Animal Care and Use Committee and were approved by the ethics committee. For the diet treatment, JAZF1-Het and JAZF1-Cont mice were divided into two groups and fed the ND (Feedlab, Guri-si, Korea) or the HFD, containing 60% fat on a caloric basis (Research Diets, New Brunswick, NJ) for 8 weeks. Mice were housed in a controlled environment with alternating 12-h light and dark cycles and free access to food and water. Bodyweight and food intake were measured every week during the entire experiment period. Unless otherwise indicated, all animal experiments were performed using male mice.

### Histological analysis

Paraffin sections (5-μm-thick) were deparaffinized with xylene and rehydrated with graded ethanol. Deparaffinized epididymal white adipose tissue (eWAT), subcutaneous white adipose tissue (scWAT), and liver sections were stained with hematoxylin and eosin (H&E), using standard histological techniques [33]. For analysis of adipocyte diameters and nuclei numbers from eWAT and scWAT, 3–8 random microscopic field (20× objective) images were taken from each mouse using Olympus BX51 (Olympus, Tokyo, Japan). Immunohistochemistry (IHC) staining was performed to examine the macrophage and monocyte cell infiltration. Deparaffinized and rehydrated eWAT and liver paraffin sections were treated with a 10-mM citrate buffer (pH 6.0) for antigen retrieval. The primary antibody was incubated overnight at 4 °C (rabbit anti-CD68, 1:100; ThermoFisher). The staining was visualized using the EnVision™ Detection System (Dako, Glostrup, Denmark). For analysis of CD68 positive cells from eWAT and liver, five–eight random microscopic field (20× objective) images were taken from each mouse using Olympus BX51.

### Body composition analysis

eWAT, scWAT, BAT, and liver were carefully dissected after the mice were sacrificed. The tissues were weighed and normalized to the whole body weight (BW) to quantify body mass. Additionally, total body fat and lean mass compositions were also assessed using a microcomputed tomography (CT) system (Quantum FX; PerkinElmer, Waltham, MA).

### Indirect calorimetry analysis

The volume of oxygen consumption (VO_2_), carbon dioxide production (VCO_2_), RER, heat production, and energy expenditure were measured using an indirect calorimetry system (Oxymax; Columbus Instruments, Columbus, OH). Mice were adapted for 3 days in a metabolic cage before recording, and data was collected for 24 h.

### Glucose tolerance test and insulin tolerance test

GTT and ITT were performed as described previously [34]. Briefly, male and female mice were fasted overnight (16 h) before the GTT. After fasting, the mice were intraperitoneally injected (i.p.) with glucose (1.5 g/kg BW). Blood glucose level was determined by tail bleeds at 0, 15, 30, 60, and 120 min using the Accu-Chek ® glucometer (Roche Diagnostics, Basel, Switzerland). The ITT was done similarly to the GTT experiment, except that the mice fasted for only 8 h and insulin (2 U/Kg bodyweight) was administered by ip injection.

### Measurement of serum insulin levels

According to the manufacturer’s instructions, the serum insulin levels were measured using a commercial mouse insulin Enzyme-Linked Immunosorbent Assay (ELISA) kit (ALPCO Diagnostic, Salem, NH).

### Insulin signaling experiment

The mice were fasted for 15 h to investigate the insulin signaling and were then intraperitoneally injected with bovine insulin (2 U/Kg) or phosphate-buffered saline (PBS). Ten minutes later, eWAT, and liver were isolated and homogenized using a homogenizer (IKA, Japan). The tissue lysates were stored at − 80 °C until the further experiment.

### Blood biochemical analysis

Blood samples were obtained by retroorbital bleeding. Samples were collected in blood collection tubes and were allowed to clot at room temperature for 2 h. Each clotted blood sample was centrifuged at 2000×*g* and 4 °C for 15 min and stored at − 80 °C until the further experiment. Biochemical analysis was performed using an automatic biochemical analyzer (PerkinElmer, Waltham, MA).

### Adipocyte differentiation in vitro

The 3T3-L1 preadipocytes were maintained in DMEM (Gibco, Waltham, MA) containing 10% calf serum (Gibco, Waltham, MA). The 3T3-L1 preadipocytes were cultured in DMEM containing 10% fetal bovine serum (FBS; Gibco, Waltham, MA) until they reached confluence to induce adipogenic differentiation. At confluence, the cells were treated with MDI (methylisobutylxanthine, dexamethasone, insulin) induction medium [DMEM containing 10% FBS, 1 μg/ml insulin, 10 μM dexamethasone, and 0.5 mM 3-isobutyl-1-methylxanthine (all from Sigma, St. Louis, MO)]. After 2 days, the cells were maintained in an insulin medium (DMEM containing 10% FBS and 1 μg/ml insulin) for 6 days. The medium was replaced every 2 days.

MEFs were isolated from E13.5d mice embryos under a dissecting microscope. MEFs were maintained in DMEM containing 10% FBS and a nonessential amino acid solution (Gibco). For inducing adipogenic differentiation, MEFs were cultured in DMEM containing 10% FBS, until they reached confluence. At confluence, the cells were treated with the DMI induction medium [MEM-α (Gibco) containing 10% FBS, 5 μg/ml insulin, 1 μM dexamethasone, and 0.5 mM 3-isobutyl-1-methylxanthine] supplemented with 5 μM rosiglitazone (Sigma). After 2 days, the cells were cultured in an insulin medium (MEM-α containing 10% FBS and 5 μg/ml insulin) supplemented with 5 μM rosiglitazone for 8–10 days. The medium was replaced every 2 days.

### Adipose tissue fractionation

eWAT from wild-type C57BL/6 mice was digested using collagenase. Adipocytes and SVFs were fractionated by centrifugation as previously described [35].

### Oil red O staining

Lipid accumulation in differentiated adipocytes from MEFs was determined by the Oil red O staining method [36]. The Oil red O staining was quantified using a spectrophotometer (PerkinElmer, Waltham, MA) at 550 nm wavelength, with 650 nm wavelength as a reference.

### Quantitative RT-PCR

Total RNA was isolated from cells and tissues, as described previously [37]. Reverse transcription was performed using PrimeScript™ 1st Strand cDNA Synthesis Kit (TaKaRa Bio, Kusatsu, Japan) following the manufacturer's instruction. Quantitative real-time PCR (qRT-PCR) was performed using SYBR Green I (TaKaRa Bio) and analyzed on a StepOnePlus™ Real-Time PCR System (Applied Biosystem, Foster City, CA). The oligonucleotide primer sets for qPCR are given in Additional file 1: Table S2. All data sets of relative mRNA expression levels were calculated based on the 2^*−∆∆*CT^ method and normalized to the glyceraldehyde-3-phosphate dehydrogenase (GAPDH) gene.

### Western blotting

Total proteins were extracted from cells and tissues using the Pro-Prep lysis buffer (iNtRON Biotechnology, Seoul, Korea), as described previously [37]. Western blotting was performed using antibodies to JAZF1(Abcam), PPARγ (Santa Cruz), C/EBPβ (Santa Cruz), *phospho*-AKT (p-AKT; Cell signaling, Beverly, MA), total-AKT (t-AKT; Cell signaling), FABP4 (Santa Cruz), and β-Actin (Santa Cruz) as primary antibodies. Immunoblots were visualized using an ECL detection kit (GE Healthcare, Piscataway, NJ).

### Statistical analysis

All data were expressed as the mean ± standard error of the mean (SEM). Statistically significant differences among the different experimental groups were determined using Student's *t*-test, with *p* < 0.05 being considered statistically significant.

## Supplementary Information

**Additional file 1: Figure S1.** Human JAZF1 mRNA expression in adipose tissue on three GEO datasets (GSE2508, GSE9624, and GSE16415). **Figure S2.** Generation of heterozygous JAZF1 deletion mice. **Figure S3.** Histological and molecular analysis of liver in JAZF1-Het and JAZF1-Cont mice. **Figure S4.** Serum analysis in JAZF1-Het and JAZF1-Cont mice. **Figure S5.** Insulin signaling and glucose homeostasis-related gene analysis in various tissues of JAZF1-Het mice. **Figure S6.** Histological and molecular analysis in the liver of JAZF1-Het-HFD and JAZF1-Cont-HFD mice. **Figure S7.** Serum analysis in JAZF1-Het-HFD and JAZF1-Cont-HFD mice. **Figure S8.** Insulin signaling and glucose homeostasis-related gene analysis in various tissues of JAZF1-Het-HFD and JAZF1-Cont-HFD mice. **Table S1.** Mouse primer sequence for allele-specific genotyping. **Table S2.** Mouse primer sequences for qRT-PCR.

## Data Availability

All data in this study are available upon request.

## Abbreviations

AUC: Area under the curve
BAT: Brown adipose tissue
BMI: Body mass index
BW: Body weight
C/EBPα: CCAAT-enhancer-binding protein α
EE: Energy expenditure
eWAT: Epididymal white adipose tissue
GTT: Glucose tolerance test
HFD: High-fat diet
ITT: Insulin tolerance test
JAZF1: Juxtaposed with another zinc finger protein 1
MCE: Mitotic clonal expansion
MEFs: Mouse embryonic fibroblasts
ND: Normal diet
NEFA: Nonesterified fatty acids
PPARγ: Peroxisome proliferator-activated receptor γ
RER: Respiratory exchange ratio
SAT: Subcutaneous adipose tissue
scWAT: Subcutaneous white adipose tissue
SVFs: Stromal vascular fractions
T2DM: Type 2 diabetes mellitus
TG: Triglyceride triglyceride
VAT: Visceral adipose tissue
VCO_2_: Volume of carbon dioxide production
VO_2_: Volume of oxygen consumption
