# Supplementary material for: JAZF1 heterozygous knockout mice show altered adipose development and metabolism
Source: Cell Biosci. 2021 Aug 19;11:161. doi: 10.1186/s13578-021-00625-1 (PMC8375039; doi:10.1186/s13578-021-00625-1)
Supplement: Supplementary file 1 — Additional file 1: Figure S1. Human JAZF1 mRNA expression in adipose tissue on three GEO datasets (GSE2508, GSE9624, and GSE16415). Figure S2. Generation of heterozygous JAZF1 deletion mice. Figure S3. Histological and molecular analysis of liver in JAZF1-Het and JAZF1-Cont mice. Figure S4. Serum analysis in JAZF1-Het and JAZF1-Cont mice. Figure S5. Insulin signaling and glucose homeostasis-related gene analysis in various tissues of JAZF1-Het mice. Figure S6. Histological and molecular analysis in the liver of JAZF1-Het-HFD and JAZF1-Cont-HFD mice. Figure S7. Serum analysis in JAZF1-Het-HFD and JAZF1-Cont-HFD mice. Figure S8. Insulin signaling and glucose homeostasis-related gene analysis in various tissues of JAZF1-Het-HFD and JAZF1-Cont-HFD mice. Table S1. Mouse primer sequence for allele-specific genotyping. Table S2. Mouse primer sequences for qRT-PCR. [file 13578_2021_625_MOESM1_ESM.pdf]

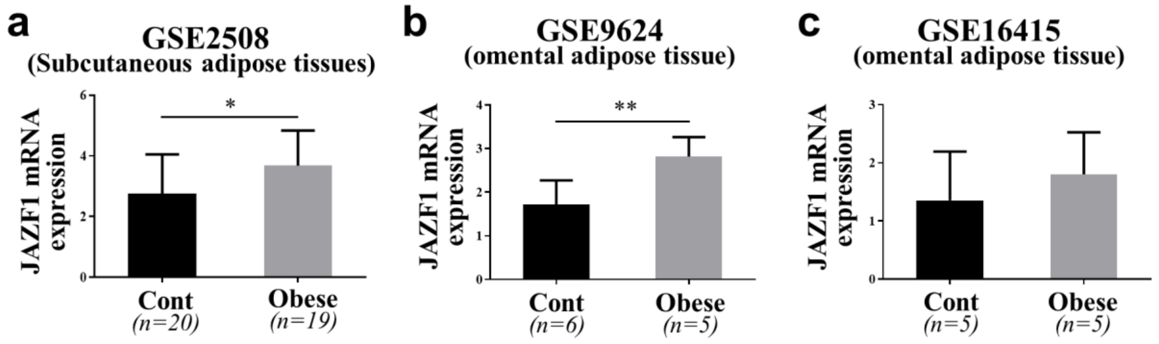

Figure S1. Human JAZF1 mRNA expression in adipose tissue on three GEO datasets (GSE2508, GSE9624, and GSE16415). a JAZF1 mRNA expression in subcutaneous adipose tissues from normal control (n = 20) and obese patient (n = 19) subjects. The data set included male and female subjects. **b** JAZF1 mRNA expression in omental adipose tissue from normal weight (n = 6) and an obese child (n = 5). The data set included male and female subjects. **c** JAZF1 mRNA expression in omentum visceral adipose tissue from normal control (n = 5) and obese subjects (n = 5). The data set included female subjects only. All data are presented as mean ± SD. *p < 0.05 and **p < 0.001. GEO, gene expression omnibus.

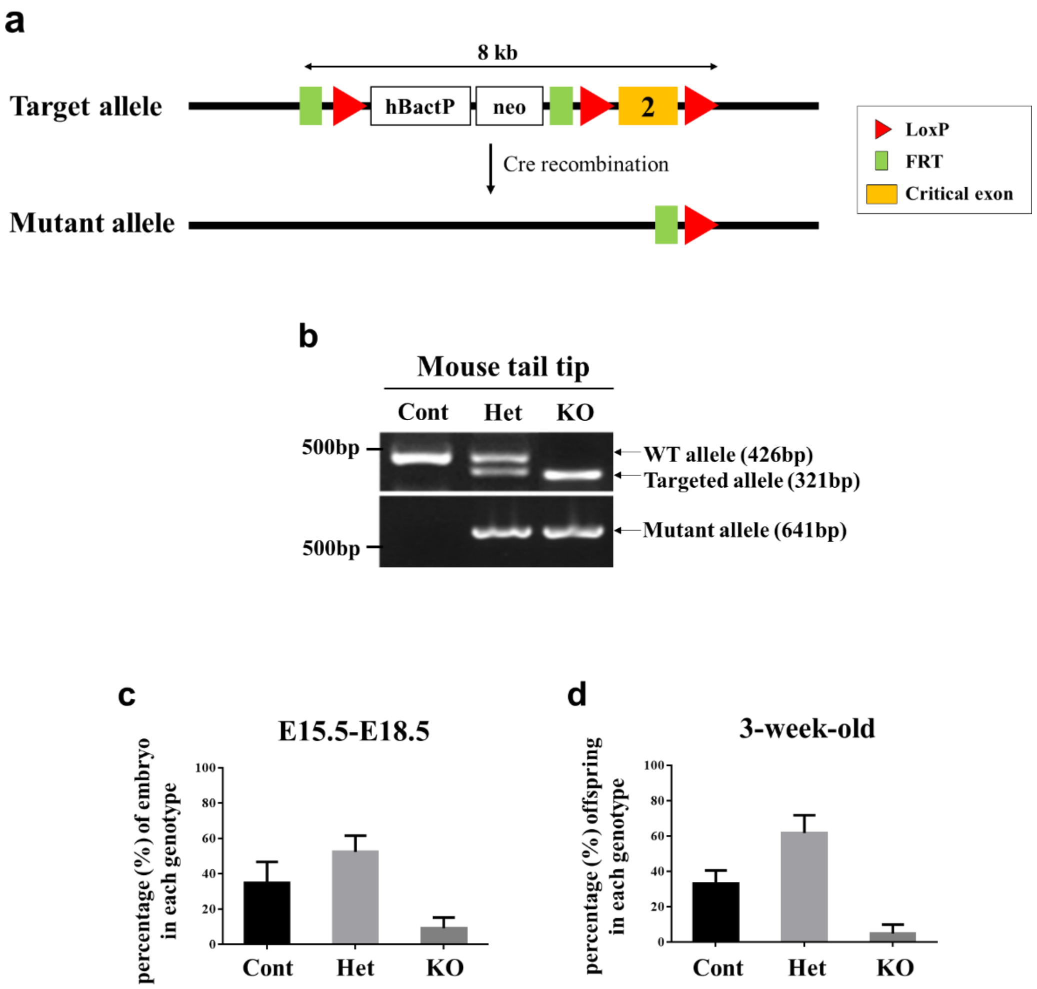

Figure S2. Generation of heterozygous JAZF1 deletion mice. a Schematic representation of the procedure for targeted JAZF1 and deleted locus lacking critical exon 2 by CMV-Cre-mediated recombination. **b** Allele-specific genotype analysis in the DNA sample from the tail tip of JAZF1 -KO (KO), JAZF1 -Het (Het), and JAZF1-Cont (Cont) mice. **c** Percentage (%) of embryos of each genotype at embryonic stages E15.5d-E18.5d by crossing male and female JAZF1-Het mice (total embryos, n = 31). **d** Percentage (%) of the offspring of each genotype obtained by crossing male and female JAZF1-Het mice (total offspring, n = 42).

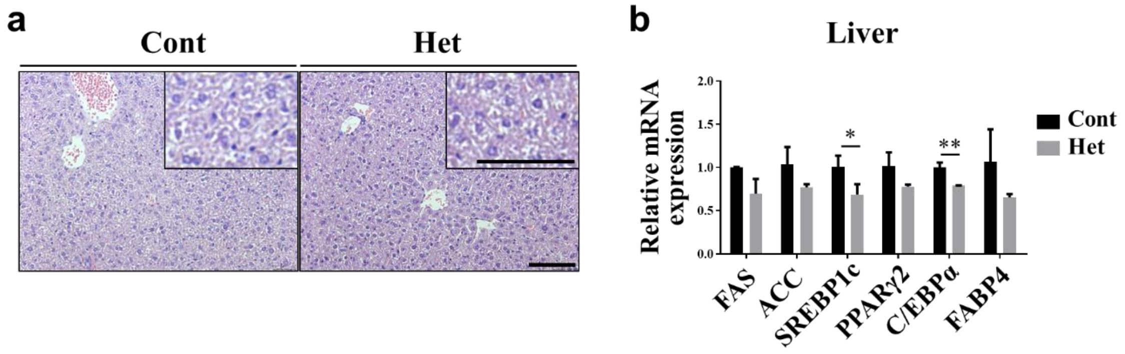

Figure S3. Histological and molecular analysis of liver in JAZF1-Het and JAZF1-Cont mice. JAZF1-Het (Het) and JAZF1-Cont (Cont) mice were fed ND for 8 weeks starting at 8-weeks-old. After 8 weeks of ND feeding, **a** H&E staining was performed in the liver of JAZF1-Het and JAZF1-Cont mice. Scale bar, 100 pm. **b** Relative mRNA expression of lipogenic markers (FAS, ACC, SREBP1c, PPARγ2, C/EBPα, and FABP4) in the liver of JAZF1-Het and JAZF1-Cont mice fed ND for 8 weeks (n = 6). All data are presented as mean ± SEM. *p < 0.05 and **p < 0.01..

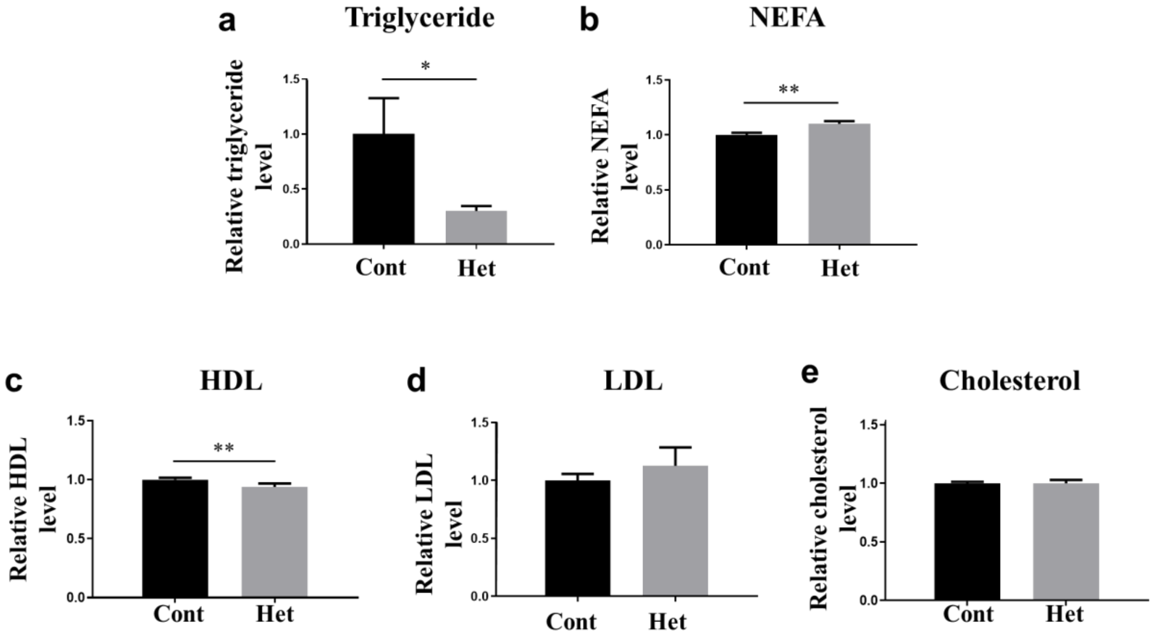

Figure S4. Serum analysis in JAZF1-Het and JAZF1-Cont mice Comparison of relative serum levels of **a** triglyceride (TG), **b** nonesterification free fatty acid (NEFA), **c** HDL, **d** LDL, and **e** cholesterol in JAZF1-Het (Het) and JAZF1-Cont (Cont) mice fed ND for 8 weeks (n = 6). All data are presented as mean ± SEM. *p < 0.05 and **p < 0.01.

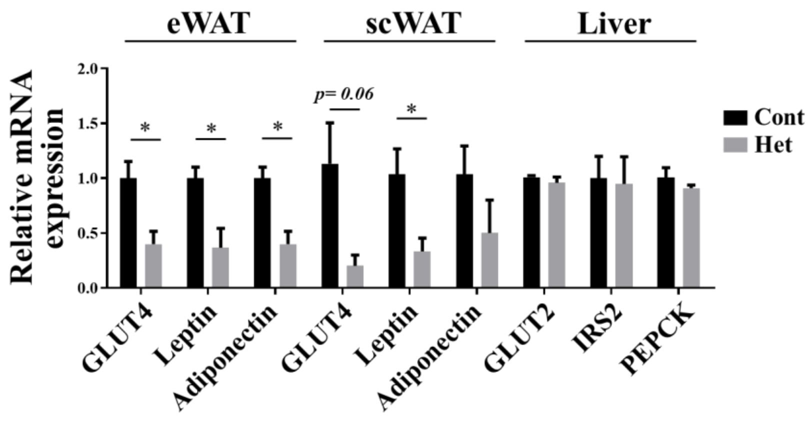

Figure S5. Insulin signaling and glucose homeostasis-related gene analysis in various tissues of JAZF1-Het mice. JAZF1-Het (Het) and JAZF1-Cont (Cont) mice were fed ND for 8 weeks starting at 8-weeks-old. After 8 weeks of ND feeding, insulin signaling and glucose homeostasis-related genes (GLUT4, GLUT2 Leptin, Adiponectin, IRS2, and PEPCK) were analyzed in various tissues (eWAT, scWAT, and liver) in JAZF1-Het and JAZF1-Cont mice (n = 6). All data are presented as mean ± SEM. *p < 0.05. eWAT, epididymal white adipose tissue; scWAT, subcutaneous adipose tissue.

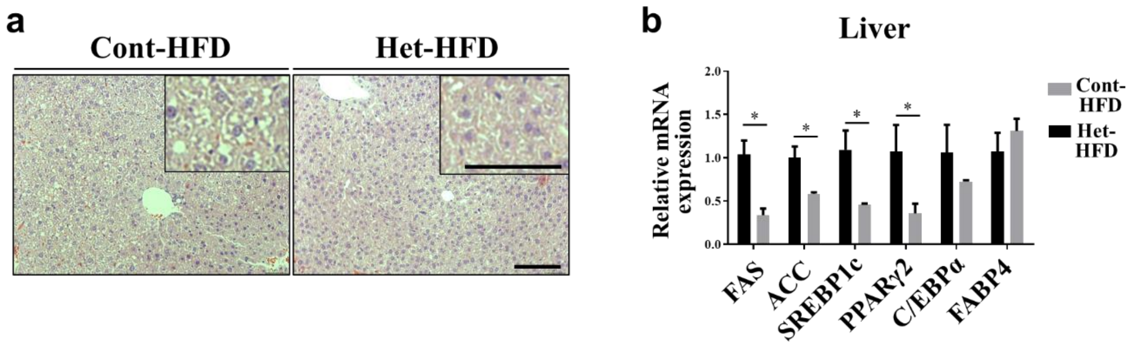

Figure S6. Histological and molecular analysis in the liver of JAZF1-Het-HFD and JAZF1-Cont-HFD mice. JAZF1-Het-HFD (Het-HFD) and JAZF1 -Cont-HFD (Cont-HFD) mice were fed HFD for 8 weeks starting at 8-weeks-old. After 8 weeks of HFD feeding, **a** H&E staining was performed in the liver of JAZF1-Het-HFD and JAZF1-Cont-HFD mice. Scale bar, 100 pm. **b** Relative mRNA expression of lipogenic markers (FAS, ACC, SREBP1c, PPARγ2, C/EBPα, and FABP4) in the liver of JAZF1-Het-HFD and JAZF1 -Cont-HFD mice (n = 6). All data are presented as mean ± SEM. *p < 0.01.

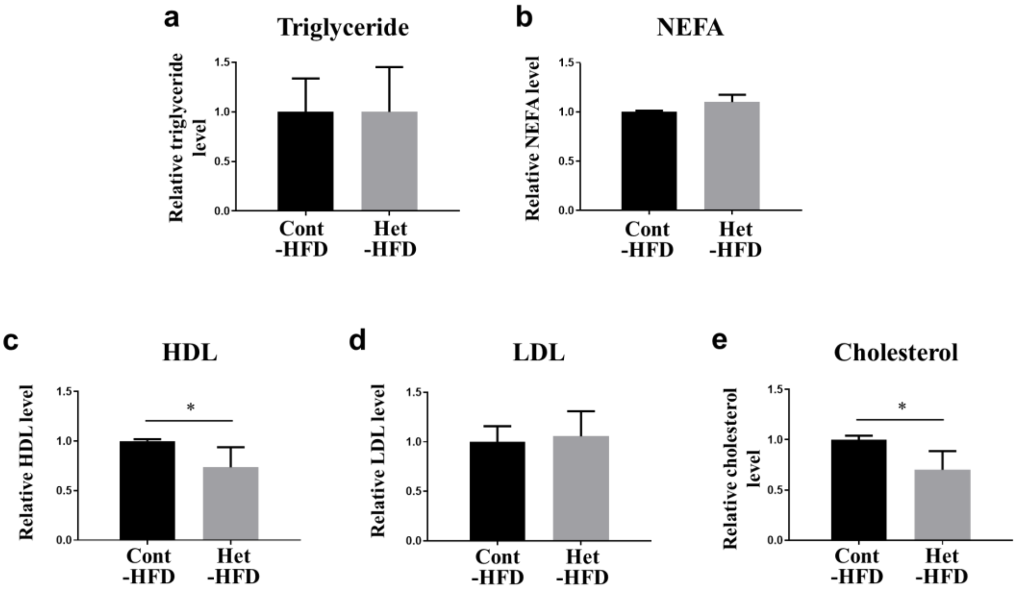

Figure S7. Serum analysis in JAZF1-Het-HFD and JAZF1-Cont-HFD mice Comparison of relative serum levels of **a** triglyceride (TG), **b** NEFA, **c** HDL, **d** LDL and **e** cholesterol in JAZF1-Het-HFD (Het-HFD) and JAZF1-Cont (Cont-HFD) mice fed HFD for 8 weeks (n = 6). All data are presented as mean ± SEM. *p < 0.05.

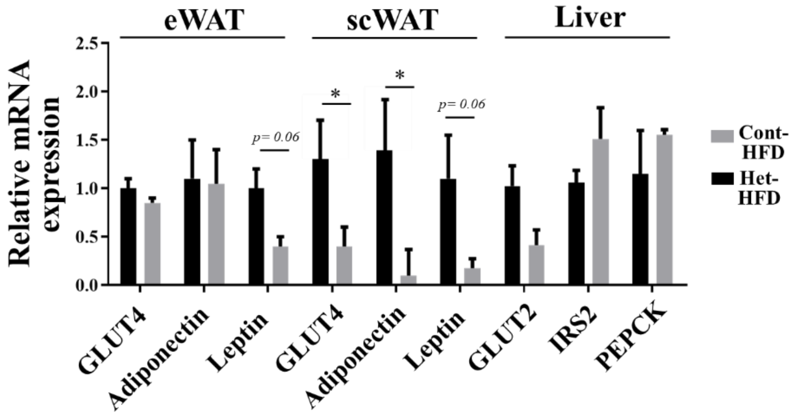

Figure S8. Insulin signaling and glucose homeostasis-related gene analysis in various tissues of JAZF1-Het-HFD and JAZF1-Cont-HFD mice. JAZF1-Het-HFD (Het-HFD) and JAZF1-Cont-HFD (Cont-HFD) mice were fed HFD for 8 weeks starting at 8-weeks-old. After 8 weeks of HFD feeding, insulin signaling and glucose homeostasis-related genes (GLUT4, GLUT2 Leptin, adiponectin, IRS2, and PEPCK) were analyzed in various tissues (eWAT, scWAT, and liver) in JAZF1-Het-HFD and JAZF1-Cont-HFD mice (n = 6). All data are presented as mean ± SEM. *p < 0.05. eWAT, epididymal white adipose tissue; scWAT, subcutaneous adipose tissue.

Table S1. Mouse primer sequence for allele-specific genotyping

Allele	Primer sequences (5'-3')	
**WT**	**5′-arm-forward**	: AGGCCTCCTCTTGACTCTGCGTGG
	**3′-arm-reverse**	: TATGTAATGGAGTGGCCTTCTAGC
**Target**	**5′-arm-forward**	: AGGCCTCCTCTTGACTCTGCGTGG
	**LAR3-reverse**	: CAACGGGTTCTTCTGTTAGTCC
**Mutant**	**Cre-forward**	: CGGTCGCTACCATTACCAGT
	**Cre-reverse**	: AACTGATGGCGAGCTCAGACC

Table S2. Mouse primer sequences for qRT-PCR

Gene	Primer sequences (5'-3')
**JAZF1**	F: CGCCGAGAACAGGAATCTCT
	R: GCTGAGGTGGAGTGGACACA
**PPARγ2**	F: CACAGAGATGCCATTCTGGC
	R: GGCCTGTTGTAGAGCTGGGT
**C/EBPα**	F: GGGCTCCTAATCCCTTGCTT
	R: CTCCATGAACTCACCCAGGAA
**C/EBPβ**	F: GTTTCGGGACTTGATGCAATC
	R: AACAACCCCGCCAGGAACAT
**FABP4**	F: AGTGAAAACTTTGATGATTATATG
	R: CCATGCCAGCCACTTTCCT
**GATA2**	F: ACAGGCCACTGACCATGAAG
	R:TCCTCGAAACATTCAGCCCC
**GATA3**	F: TTGGAATGCAGACACCACCT
	R: AGGAGTCTCCAAGTGTGCGAA
**KLF2**	F: AAGAGCTCGCACCTAAAGGC
	R: CTTTCGGTAGTGGCGGGTAA
**KLF3**	F: ACTCACGGGATACAGGTGGA
	R: GTGGGACGGGAACTTCAGAG
**GLUT4**	F: CTGGCCCCATCCCCTGGTTCA
	R: CAAATGTCCGGCCTCTGGTTTCAG
**GLUT2**	F: GGCCCTTGTCACAGGCATTCTTAT
	R: TGGACAGAAGAGCAGTAGCAGACA
**PEPCK**	F: TGCGGATCATGACTCGGATG
	R: AGGCCCAGTTGTTGACCAAA
**Adiponectin**	F: CCTCTTAATCCTGCCCAGTCA
	R: GCCATCCAACCTGCACAAGT
**Leptin**	F: ATCTCCGAGACCTCCTCCATC
	R: CATCCAGGCTCTCTGGCTTCT
**IRS2**	F: TCTTTCACGACTGTGGCTTCCTT
	R: CACTGGAGCTTTGCCCTCTGC
**FAS**	F: TGGTGGGTTTGGTGAATTGTC
	R: GCTTGTCCTGCTCTAACTGGAAGT
**ACC**	F: ATGTCCGCACTGACTGTAACCA
	R: TGCTCCGCACAGATTCTTCA
**SREBP1c**	F: GATCAAAGAGGAGCCAGTGC
	R: TAGATGGTGGCTGCTGAGTG
**18S rRNA**	F: GTAACCCGTTGAACCCCATT
	R: CCATCCAATCGGTAGTAGCG
**JAZF1***	F: CTCCACCTCGACATAGCAGT
	R: TCCTGATCATCTCGGCAGAC
**β-actin***	F: AGGGAAATCGTGCGTGACAT
	R:TGCTAGGAGCCAGAGCAGTA

*semiquantitative RT-PCR primers
